# Growth Hormone Deficiency Is Frequent After Recent Stroke

**DOI:** 10.3389/fneur.2018.00713

**Published:** 2018-09-06

**Authors:** Thomas Lillicrap, Carlos Garcia-Esperon, Frederick Rohan Walker, Lin Kooi Ong, Michael Nilsson, Neil Spratt, Christopher R. Levi, Mark Parsons, Jörgen Isgaard, Andrew Bivard

**Affiliations:** ^1^Department of Neurology, John Hunter Hospital, University of Newcastle, Newcastle, NSW, Australia; ^2^Hunter Medical Research Institute, University of Newcastle Newcastle, NSW, Australia; ^3^Department of Internal Medicine, Sahlgrenska Academy at the University of Gothenburg, Gothenburg, Sweden

**Keywords:** ischemic stroke, growth hormone, neuro recovery, rehabilitation, cognition

## Abstract

**Introduction:** The incidence of pituitary dysfunction after severe ischemic stroke is unknown, however given the increasing attention to pituitary dysfunction after neurological injuries such as traumatic brain injury, this may represent a novel area of research in stroke.

**Methods:** We perform an arginine and human growth hormone releasing hormone challenge on ischemic stroke patients within a week of symptom onset.

**Results:** Over the study period, 13 patients were successfully tested within a week of stroke (baseline NIHSS 10, range 7–16). Overall, 9(69%) patients had a poor response, with 7(54%) of these patients meeting the criteria for had human growth hormone deficiency. Other measures of pituitary function were within normal ranges.

**Conclusion:** After major ischemic stroke, low GH levels are common and may play a role in stroke recovery.

## Introduction

Enhancement of recovery after ischemic stroke has been a challenging area, with no intervention being widely adopted to promote recovery in clinical practice. In the search for innovative treatment targets post stroke, we decided to investigate pituitary function after stroke with a focus on human growth hormone (hGH). Other acute brain insults such as traumatic brain injury (TBI) and subarachnoid hemorrhage have demonstrated hypopituitarism in around one quarter of patients shortly after injury (1). Although TBI has previously been considered as a rare cause of hypopituitarism, an increased prevalence of neuroendocrine dysfunction in patients with TBI has been reported over the last 15 years as endocrinological testing has become more common in this patient group. The symptoms of hypopituitarism depend on which hormone is deficient, but for hGH, they include impaired attention and memory, reduced energy, cognitive dysfunction, a decrease in muscle mass and strength, reduced bone mass and density and cardiac dysfunction, all of which are common in stroke survivors (2). We hypothesized that patients with a severe ischemic stroke resulting in significant residual disability would experience hypopituitarism, particularly affecting hGH secretion. The aim of this study was to investigate the potential incidence of hGH deficiency from 7 days after stroke onwards.

## Methods

Consecutive patients presenting to the John Hunter Hospital Emergency Department with first ever clinical diagnosis of an ischemic stroke were assessed for this study. The acute clinical diagnosis was confirmed by multi modal computed tomography (CT) which included CT angiography, CT perfusion and a noncontrast CT. Patients presenting with symptoms typical of transient ischemic attack or stroke mimics such as migraine with aura, seizures, acute confusional states, hypoglycaemia, diabetes, or patients with symptoms related to known prior stroke, or patients with a known history of conversion disorder were excluded from the study.

Baseline clinical data recorded included demographics (age, sex), vascular risk factors (hypertension, diabetes mellitus, dyslipidemia, atrial fibrillation, smoking history, prior stroke/TIA, and ischemic heart disease), clinical features such as the baseline and 24 h National Institutes of Health Stroke Scale (NIHSS), and premorbid modified Rankin Score (mRS), mRS at discharge and 90 days post stroke.

If the patient consented to the study, researchers would organize a fasting blood assessment for the next morning to be taken between 8 and 10 a.m. All enrolled patients underwent growth hormone (GH) provocative test with a GH-releasing-hormone (GHRH) and arginine infusion (3). GH reserve was assessed by GHRH (1 μg/kg bolus) plus arginine (ARG; 0.5 g/kg iv up to maximum of 30 g in 60 ml saline, infused over a 30 min period) testing. A GH response peak < 11 μg/liter in patients with BMI < 25 kg/m2, < 8 μg/liter in patients with BMI between 25 and 30 kg/m2, and < 4.2 μg/liter in those with BMI 30 kg/m2 or greater was considered diagnostic for GHD (3). From the initial 10 mL sample we will also test the levels of serum insulin-like growth factor 1 (IGF-1), thyroid function with thyroid specific hormone (TSH) and T1 and T2 and cortisol. In addition to the GH test, participants were tested for levels of TSH, T3, T4, ACTH, LH, testosterone, FSH, and cortisol. These tests were processed and analyzed by the local pathology department on the same day as the GH test.

If the patient was able, study specific assessments were also performed at the time of blood testing and included the Montreal Cognitive Assessment (MoCA) and the NIHSS.

## Results

From July 2016 to July 2017, 13 patients were enrolled in this prospective study. Median time of testing was 7 days post stroke (range 4–10, Table 1) and median age was 71 (range 54–78). Median hospital admission National Institutes of Health Stroke Scale (NIHSS) was 10 (range 7–16). Seven patients had middle cerebral artery stroke, two had lacunar infarction, two patients had posterior circulation stroke, and two had brain stem stroke. The mean 24 h post stroke MRI DWI lesion was 55 mL (range 8–92 mL). The median 90 day modified Rankin Score median was 2 (range 1–4). Of the 13 patients, 7 (54%) fulfill criteria for hGH deficiency when the GH peak level was related to BMI and two patients had a borderline response (mean abnormal GH peak 4.4, Table 2). In the 8 included men, 6 showed low levels of testosterone below the normal reference range (mean low testosterone result was 3.2 nmol/L), and two showed normal levels (mean normal testosterone result was 12.8 nmol/L). Lastly, only one of the studies 5 females showed low levels of luteinising hormone (LH). FSH was elevated in 8 patients (mean abnormal FSH level 46.3, reference range 1–12) as can be expected in elderly subjects. Insulin-like growth factor 1 (IGF-1) was mostly within the normal range in the tested patients, however two patients had low IGF-1 concomitant with GH deficiency. TSH, T3, T4, and cortisol were all within normal reference ranges.

**Table 1:** Individual patient data from the study.

**Patient number**	**Age range**	**BMI**	**Days since stroke**	**Acute NIHSS**	**24 h NIHSS**	**Stroke territory**	**FU infarct volume**	**Pre- stroke mRS**	**Discharge mRS**	**Discharge destination**	**90-day mRS**
1	75–80	28.8	9	8	7	R POCI	8	0	4	Rehabilitation	1
2	75–80	24.6	8	10	3	R MCA	22	0	1	Home	1
3	75–80	25.5	9	16	17	R MCA	95	0	5	Rehabilitation	4
4	70–75	30.5	6	8	8	R MCA	73	0	4	Rehabilitation	3
5	70–75	27.1	4	10	10	R MCA	63	0	5	Rehabilitation	2
6	65–70	20.5	6	18	2	R MCA	82	0	4	Home	1
7	50–55	31	9	2	1	Bilat Cerebellum	67	0	3	Rehabilitation	3
8	65–70	27	7	26	23	L ICA, MCA	51 (HT1)	0	4	Rehabilitation	3
9	75–80	40.6	9	2	2	Bilat Cerebellum	12	1	4	Rehabilitation	2
10	75–80	31.1	4	20	3	R MCA	92	0	4	Rehabilitation	3
11	70–75	23.2	10	2	2	R POCI	18	1	1	Home	1
12	55–60	26.0	10	2	1	L LAC	19	0	1	Home	1
13	75–80	22.0	6	20	7	R MCA	90 (HT1)	0	3	Rehabilitation	2

**Table 2:** Pituitary function results from the study.

**Patient number**	**Peak GH ug/L**	**BMI**	**GH Deficient (corrected for BMI)**	**IGF-1 (ug/L, reference range 55–186)**	**Cortisol (nmol/L, reference range 101–535)**	**FSH (IU/L, reference range 1–12)**	**TSH (mU/L, reference range 0.4–5)**	**T4 (pmol/L, reference range 10–20)**	**T3 (pmol/L, reference range 2.3–5.7)**	**Testosterone (nmol/L, reference range 9–38)**	**Luteinizing hormone (IU/L, reference range – Males 0.6–12.1, females 5.2–62)**
1	4.16	28.8	Yes	115	249	2.7	1.57	13.2	3.9	4.3	8.6
2	36.30	24.6	No	191	328	13.3	0.7	12.5	4	3.3	1.3
3	4.70	25.5	Yes	108	350	2.3	1.05	17.5	3.7	6.4	1.5
4	2.10	30.5	Yes	56	472	2.7	0.91	13.1	4.4	13.4	5.2
5	7.90	27.1	Borderline	149	421	57.6	2.16	17	2	N/A	3.2
6	40.00	20.5	No	202	511	76.4	1.08	13.4	3.6	N/A	27.5
7	4.53	31	Yes	178	470	77.8	2.6	12	3.4	N/A	27.3
8	1.60	27	Yes	158	433	1.6	2.47	13	2.7	7	0.6
9	26.73	40.6	No	201	238	55.8	2.25	10.9	1.8	N/A	24.2
10	18.03	31.1	No	126	278	36.8	3.89	12.6	4.5	N/A	14.9
11	9.93	23.2	Yes	60	404	39.9	1.41	12.2	5.1	12.2	13.1
12	6.97	26.0	Borderline	125	358	1.3	2.22	11.4	4.2	0.8	< 0.2
13	3.40	22.0	Yes	51	488	12.8	3.53	11	2.4	4.1	4.2

*Red indicates the results which are outside of the normal reference ranges and yellow indicates borderline results*.

Only 5 of the 13 enrolled patients were able to complete cognitive assessments, with those unable to complete the assessment due to stroke deficits. In these 5 patients, the Montreal Cognitive Assessment was low (median 22, range 19–24, normal score 26). All patients with GH deficiency were unable to complete the MoCA assessments.

## Discussion

In a cohort of patients with a severe ischemic stroke, we report that there is a considerable number of patients who fulfill criteria for GH deficiency after a provocative test (54–69%) in the first month after stroke. There are limited case series documenting early growth hormone dysfunction after stroke, however, reported pituitary dysfunction may last for up to 12 months (4). This study used the gold standard growth hormone with arginine stimulation test to assess hGH levels, which is challenging in routine practice and may explain why hGH testing is not commonly performed. The presence of GH deficiency in our study was more common than previously reported (4). However, one important difference between this and our study was that Bondanelli and co-workers performed endocrine testing later and within larger time span (1–3 month after stroke and then after 12–15 months) which may contribute to differences in results.

The biological reason behind the observed decline in hGH after stroke, TBI, or subarachnoid hemorrhage is largely unknown. Importantly, pituitary dysfunction after TBI is associated with a worse neurological outcome and increased morbidity and mortality (5), which may also be relevant to stroke survivors or future study outcomes. Animal studies have indicated that GH treatment after stroke accelerates physical recovery (6) and leads to better learning and memory (7). Importantly, GH supplementation has also been shown to reduce neuronal loss in animals, which is attributed to either neuroprotection or stimulation of neurogenesis (8). Previous human trials of hGH treatment in a deficient clinical population of TBI patients of a comparable age range has been shown to result in significantly improved cognitive outcomes. (9, 10) GH receptors are expressed in many regions of the brain, (11) but importantly those expressed in the hippocampus (12) may help explain the cognitive enhancement of GH therapy and may represent a viable intervention in the future.

In conclusion, we report a novel finding of GH deficiency after ischemic stroke, which may ultimately represent a novel treatment to enhance post stroke cognition and recovery. Further studies in stroke survivors are required to understand if those with hGH deficiencies or any other pituitary dysfunction have poorer recovery, and what factors predict which patients are likely to experience hGH deficiency after stroke. Moreover, further studies following endocrine function over time in patients after stroke may be required since pituitary deficiency may be transient in some patients.

## Ethics statement

This study were approved by the Hunter New England Human Research Ethics Committee and each participant provided written informed consent.

## Author contributions

TL: acquisition of data, critical revision of the manuscript for important intellectual content and study supervision; CG-E: acquisition of data, critical revision of the manuscript for important intellectual content and study supervision; FW: analysis and interpretation, critical revision of the manuscript for important intellectual content and study supervision; LO: critical revision of the manuscript for important intellectual content and study supervision; MN: critical revision of the manuscript for important intellectual content and study supervision; NS: acquisition of data, analysis and interpretation, critical revision of the manuscript for important intellectual content and study supervision; CL: analysis and interpretation,critical revision of the manuscript for important intellectual content and study supervision; MP: Study concept and design, analysis and interpretation, critical revision of the manuscript for important intellectual content and study supervision; JI: critical revision of the manuscript for important intellectual content and study supervision; AB: Study concept and design, acquisition of data, analysis and interpretation, critical revision of the manuscript for important intellectual content, and study supervision.

### Conflict of interest statement

The authors declare that the research was conducted in the absence of any commercial or financial relationships that could be construed as a potential conflict of interest.
